# Factors Associated with Family Functioning During Pregnancy by Adolescent and Young Adult Women

**DOI:** 10.1089/whr.2023.0083

**Published:** 2024-04-03

**Authors:** Jie Zhong, Yzette Lanier, Audrey Lyndon, Trace Kershaw

**Affiliations:** ^1^ NYU Rory Meyers College of Nursing, New York University, New York, New York, USA.; ^2^ School of Public Health, Yale University, New Haven, Connecticut, USA.

**Keywords:** adolescent health, family health, postpartum

## Abstract

**Introduction:**

Pregnancy represents a stressful period for both women and their families. Whether the family maintains functioning during pregnancy could have significant implications on maternal and child health. In this study, we explored individual- and family-level factors associated with family functioning in adolescent and young adult mothers.

**Methods:**

This study was a secondary analysis of 295 young mothers, ages between 15 and 21 years. Multivariate logistic regression models were conducted to estimate adjusted odds ratios of exploratory factors on the risk of being in high family functioning group. The parent study was approved by the Institutional Review Boards at Yale University.

**Results:**

The mean score of family functioning was 5.14 out of 7. With the inclusion of individual-level factors (Model 1), significant associations were observed between high family functioning and having ever attended religious services (OR = 2.22, 95% CI: 1.20–4.09), low perceived discrimination (OR = 3.04, 95% CI: 1.60–5.75), and high perceived social support (OR = 3.74, 95% CI: 2.01–6.95). After including both individual- and family-level factors (Model 2), results identified significant associations between high family functioning and annual household income>$15,000 (OR = 9.82, 95% CI: 1.67–57.67, *p* = 0.011) and no experience of violence from any family members (OR = 4.94, 95% CI: 1.50–16.21, *p* = 0.008).

**Discussion:**

The models of care should be structured to support the continuity of maternity care in which health care providers have the opportunity to discover and utilize each family's strengths to provide the optimal caring experience for young mothers and their families as a unit.

## Introduction

Pregnancy represents a normative but stressful period for women and their families. Chronic stressors during pregnancy could include economic insecurity, role confusion, interpersonal difficulties, and physical demands from childcare.^1,2^ At the same time, childbearing families have the potential to face some structural stressors such as discrimination, lack of access to care, and food insecurity.^3–6^

Pregnancy can be a particularly stressful period for childbearing families with adolescent and young adult mothers. Young mothers face unique developmental challenges related to transiting to young adulthood such as the desire for self-identification.^7^ Stress related to pregnancy has been found to be associated with poor developmental outcomes for young mothers and their children.^8–10^ However, not all young mothers and their children experience poor developmental outcomes, with many young mothers managing to be resilient despite the stress of pregnancy.^8–10^ As such, they are able to adapt well to motherhood and create positive lives for themselves and their children.

Resilience is defined as the process of, capacity for, or outcome of successful adaptation despite challenging or threatening circumstances.^11^ While previous studies have primarily focused on the risks associated with the stress of pregnancy for young mothers, there is a need to explore resilience in the context of pregnancy and facilitate the adaptation to new roles across family members.

Family resilience is defined as “characteristics, dimensions, and properties of families, which help families to be resilient to disruption in the face of change and adaptive in the face of crises.”^12^ Given that individuals are embedded within families, individual stressful situations affect the whole family system, posing risks for both individual and family dysfunction. Meanwhile, abilities to cope with and adapt to stress can be nurtured and learned in a relational context, such as with the help of family members. Even though individuals sometime can perceive negative interactions and relationships with families, it is important for them to know that there may be someone in the family who can support them in developing their competence and complete personal transformation.

Family resilience may be particularly important to a pregnant woman as her family members, including her partner and biological families, may present her closest form of intimate support.^13^ Research on family resilience has been conducted in various contexts, such as families living in poverty, families of children with autism, and families dealing with prostate cancer.^14–16^ However, little research has been conducted on childbearing families. Adjustment to pregnancy, in conjunction with other demands, may create a state of crisis for some families. As such, family resilience during pregnancy remains an underexplored but significant area.

An important aspect of family resilience is family functioning. Family functioning is defined as the degree to which families can successfully fulfill their functions so that individual family members and other social systems benefit in front of stressful situations.^17^ Family functioning reflects family efforts to maintain a level of balance, harmony, and coherence when facing family stress.^18,19^ Attributes of family functioning include fulfillment of family roles, direct and clear communication, and close emotional bonding.^20–22^ High family functioning occurs within a family environment when there is clear communication, well-defined roles, cohesion, good affect regulation, and problem-solving ability.^20–22^ Studies have associated the construct of family resilience with how families can maintain high functioning.^23–25^ Therefore, family functioning can be used as one indicator of family resilience to indicate if a family is equipped with abilities to be functional in the context of stress.

To fully understand the concept of family functioning during pregnancy, there is a need to identify factors associated with family functioning. To date, limited research has examined factors related to family functioning among childbearing families, which could provide information on assessing childbearing families and give insight into how to effectively support young mothers in the face of pregnancy.^13^ Exploring factors associated with higher family functioning can provide information on the characteristics of these young mothers who perceive higher family functioning. Identifying these protective factors can help engage significant family members in the maternity care of young women as her closest form of intimate support. On the contrary, identifying childbearing families with lower family functioning would provide the opportunity to intervene early and optimize the care for young mothers living in stressful environments.

The current study aimed to explore individual- and family-level factors associated with family functioning. The specific individual- and family-level factors included in the current study were selected based on a review of the literature on factors associated with family resilience.^13,26^ On the individual level, significant factors related to high family functioning among adult mothers have been identified as older age, no alcohol during pregnancy, individual perceptions of low stress, and high interpersonal support.^13^ Additionally, based on the conceptual review of family resilient factors, how the family is constructed can be a prominent factor for resilient families.^26^ Witnessing or experiencing domestic violence indicates the dysfunction of some family relationships.^27,28^ These factors at the family level have not been explored regarding family resilience in the context of pregnancy. Therefore, this study included family-level factors of household income, household arrangement, and interpersonal violence from a partner or extended family network.

## Methods

### Procedures

This study is a secondary analysis of data obtained from the Parenting and Relationship Transition & Risk Study (PARTNRS).^29^ Participants were identified at Obstetrics & Gynecology or ultrasound clinics from four university-affiliated hospitals in urban areas of Connecticut. Both parents of the focal baby were screened with the following eligibility criteria: (1) pregnant women at greater than 23-week gestation; (2) pregnant women between 15 and 22 years of age at the time of baseline interview; (3) both women and men reported being in a romantic relationship with each other and reported being the biological parents of the unborn baby; (4) both parents agreed to participate in the study; (5) both parents were able to speak English or Spanish.

Between July 2007 and February 2011, 296 adolescent and young adult mothers and partners were enrolled in the study. Surveys were facilitated at more than 23-week gestation, 6-month postpartum, and 12-month postpartum. Written informed consent was obtained from all participants. All procedures were approved by Institutional Review Boards at Yale University. In the current study, only baseline data (23-week gestation) were utilized.

### Measures

#### Predictors

##### Individual-level factors

Demographics included age, years of education, race (Black, Latino, White, or Others), immigrant status (Born in the United States or Born outside the United States), and current employment (Not working, Part-time, or Full-time), relationship status (Single/never married, Cohabit/not married, Married, Separated), parity status (Nulliparous, Multiparous).

Years of education was measured by the questions of “What is the highest grade you completed?.” Values of 1–12 indicated 1st grade to high school. Value of 14 was given to the response of “Some college,” 16 to “Graduated college,” 18 to “Some graduate or professional school,” and 20 to “Completed graduate or professional school.” The range of years of education was between 1 and 20.

Ever attending religious services was measured using the question of “how often you go to church or temple.” Response choices include “0 = Never,” “1 = Only on major religious holidays,” “2 = A few times a year,” “3 = Once a month,” “4 = A couple times a month,” “5 = Once a week,” “6 = More than once a week.” Response distribution was skewed, showing that one third of participants chose “0 = Never” (34.5%). Therefore, the variable was dichotomized as “never attended religious services” with the response of “0 = Never” or “ever attending religious services” with the rest of responses.

The use of alcohol, cigarette, or marijuana before pregnancy was measured using the question, “how often you used alcohol/cigarette/marijuana 3 months before pregnancy.” The answer options included “never,” “rarely,” “sometimes,” “often,” and “every day.” Response distribution was skewed, showing that majority of participants chose “never” for substance use (52.9% for alcohol use, 62.7% for cigarette use, and 70.8% for marijuana use). Therefore, the variable was dichotomized as “no use of alcohol/cigarette/marijuana before pregnancy” or “use of alcohol/cigarette/marijuana before pregnancy.”

Perceived discrimination was measured using an adapted version of 20-item Daily Life Experiences-Frequency Scale.^30^ Sample items included “Being ignored, overlooked, or not given service (in a restaurant, store, etc.)?” and “Being accused of something or treated suspiciously?” The response scale ranged from “0 = Never,” “1 = Less than once a year,” “2 = A few times a year,” “3 = About once a month,” “4 = A few times a month,” and “5 = once a week or more.” In two racially/ethnically diverse samples of college and graduate students, internal consistency, split-half, and test/retest reliabilities were between 0.69 and 0.96 with 93% of the coefficients above 0.75; the pattern of construct validity correlations suggested that the scale was measuring intended constructs.^30^ The Cronbach's alpha coefficient in this sample was 0.96.

General stress was measured using the 10-item Perceived Stress Scale.^31^ Sample items included, “In the past month, how often have you felt upset by something that happened unexpectedly?” and “In the past month, how often have you felt unable to control important things in your life?” Response choices included “0 = Never,” “1 = Almost never,” “2 = Sometimes,” “3 = Fairly often,” and “4 = Very often.” Early studies found the measure had good psychometric properties. For example, the Cronbach's alpha coefficients were between 0.84 and 0.86 across two samples of college students and a community smoking cessation group.^32^ The Cronbach's alpha coefficient in this sample was 0.75.

Social support was measured using the 10-item Medical Outcomes Study Social Support Survey.^33^ Participants responded using a 5-point Likert scale ranging from “0 = None of the time” to “4 = All of the time.” Sample items included “How often is someone available to give you good advice about a crisis.” Prior studies recognized the measure as reliable and valid. For example, the Cronbach's alpha was 0.97 for a sample of high-risk pregnant women with 18–34 years of age.^34^ The Cronbach's alpha coefficient in this sample was 0.87.

##### Family-level factors

Household arrangement was measured using household size and family member coresidence. Household size was indicated by the question “other than you, how many people currently live in the house” which ranged from 0 to 30. Then participants were asked to answer, “who do you currently live with” such as partner, mother, father, and siblings.

Annual household income was measured using a single question “What is your household income (the total income before taxes earned by all members of your household) per year?.” Response choices included “0 = $0-$4,999,” “1 = $5,000-$9,999,” “2 = $10,00-$14,999,” “3-$15,000-$19,999,” “4 = $20,000-$24,999,” “5 = $25,000-$34,999,” “6 = $35,000–49,999,” “7 = $50,000 or more,” and “97 = Don't know.” Household income was coded in the cohort as “$0-$4,999,” “$5,000-$14,999,” “$15,000-$24,999,” “$25,000-$34,999,” and “≥$35,000.” Average household income of the sample was $15,471. Household income ≥$15,000 was used as the cutoff point in the current study.

Interpersonal violence was assessed using the questions from the revised Conflict Tactics Scale.^35^ Questions are related to physical hurt (shoved, slapped, punched, hit), forced sex, and verbal insult. Example questions included “Have you ever gotten any injuries when you were shoved, slapped, punched, hit, or physically hurt by father of your baby? (Yes/No)” and “Who has shoved, punched, hit, slapped, or physically hurt you? (A family member/A partner's family member/A friend/A stranger).” Participants who answered “Yes” to any types of interpersonal violence from father of the baby were labeled as a history of violence from father of the baby. Participants who chose “A family member” to any types of interpersonal violence were labeled as a history of violence from extended family network.

#### Outcome

##### Family functioning

Family functioning was measured by the 12-item Family Functioning Scale (FFS) adapted from the original 40-item FFS.^22^ The overall 12 items evaluated the general dimensions of family functioning - family communication, positive family affect, and family conflict. Participants indicated how accurately the statements describe their biological family (e.g., parents, siblings, aunts/uncles, grandparents) on a 7-point Likert scale from 1 = Never to 7 = Always. The mean score was calculated, ranging from 1 to 7. A higher mean score indicated better family functioning with efficient family communication, positive family affect, and less family conflict. The FFS reported a fair internal consistency in a multisource sample, including college students and their friends/families, clients of psychotherapies, and church members.^22^ It also showed concurrent validity, as demonstrated by correlations with the FACES III measure of family function.^22^ The Cronbach's alpha coefficient in this sample was 0.87.

### Data analyses

All data analyses were conducted using STATA Version 15.^36^ The normality of the outcome variable was tested using the Shapiro–Wilk test. Family functioning scores were right skewed. Therefore, participants were divided into two groups based on their family functioning scores: individuals who scored below and at the 25% quartile were characterized as low family functioning, and individuals who scored equal to or above the 25% quartile were characterized as high family functioning.

For descriptive analyses, frequencies and percentages were computed for categorical variables and means and standard deviations were computed for continuous variables. Bivariate analyses were conducted to identify variables that would be included in the regression models. As such, Chi-Square tests/*t* tests were used to estimate the associations between categorical/continuous variables and family functioning groups. Eight categorical variables and three continuous variables were identified for the bivariate logistic regression models. These 11 predictors that would be included in the regression models were dichotomized for a consistent interpretation on the effect of each predictor on the outcome. For three continuous variables, scores of perceived discrimination and stress were left skewed, while score of perceived social support was right skewed. Therefore, discrimination and stress were dichotomized based on 75% quartile, whereas social support was dichotomized based on 25% quartile.

Bivariate logistic regression models were conducted to examine the associations between the 11 dichotomous variables and family functioning groups, with unadjusted odds ratio (OR) and 95% confidence interval (CI) calculated to estimate the effect of each factor on the risk of high family functioning. An alpha level of 0.10 was used to determine a selection of significant variables for further multivariate models. This cutoff for inclusion was intended to ensure that factors that might become significant after adjusting for other factors would not be excluded from the analysis.^13^ A multivariate forward stepwise logistic regression analysis was constructed to estimate adjusted OR and 95% CI of included significant factors from bivariate regression models on high family functioning, first including individual-level factors and then family-level factors. Statistical significance was considered at *p* < 0.05.

## Results

The sample consisted of 295 adolescent and young adult mothers. The mean age was 18.7 (±1.63), ranging from 15 to 21. Average years of education was 11.75 (±1.82), ranging from 8 to 20. Most participants self-identified as Black (39.5%) and Latino (39.5%). Most participants reported currently not working (71.5%), and cohabit, not married (46.4%).

The mean for FFS was 5.14 (±1.05) with the range of 1.2 to 7. Results of Chi-Square tests (Table 1) showed that women living in families with high family functioning were more likely to be Black or Latino, married, ever attending religious services, reported higher annual household income, mother coresidence, sibling(s) coresidence, or no violence from a partner or extended family. Results of *t* tests (Table 2) showed that lower perceived discrimination and stress scores and higher social support scores were associated with the high family functioning group.

**Table 1.** The differences on categorical variables between low and high family functioning groups based on Chi-Square tests (*N* = 295)

Sample characteristics	Total *N* (%)	Low family functioning *N* (%)	High family functioning *N* (%)	*p*-values
Number	295	78 (26.4)	217 (73.6)	—
Race				
Black	117 (39.5)	32 (41.0)	85 (39.2)	0.087
Latina	117 (39.5)	24 (30.8)	93 (42.9)	
White	50 (16.9)	16 (20.5)	33 (15.2)	
Others	12 (4.01)	6 (7.7)	6 (2.8)	
Immigrant				
Yes	32 (10.9)	7 (9.0)	25 (11.5)	0.535
No	263 (89.2)	71 (91.0)	192 (88.5)	
Current employment				
Not working	211 (71.5)	61 (78.2)	150 (69.1)	0.205
Working part time	61 (20.7)	14 (18.0)	47 (21.7)	
Working full time	23 (7.8)	3 (3.8)	20 (9.2)	
Relationship status				
Parity				
Nulliparous	232 (78.9)	61 (78.2)	171 (79.2)	0.607
One parity	52 (17.8)	13 (16.7)	39 (18.1)	
2 or more live births	10 (3.4)	4 (5.1)	6 (2.8)	
Ever attending religious services				
Yes	193 (65.5)	43 (55.1)	150 (69.1)	0.026
No	102 (34.5)	35 (44.9)	67 (30.9)	
Alcohol use before pregnancy				
Yes	139 (47.1)	33 (42.3)	106 (48.9)	0.321
No	156 (52.9)	45 (57.7)	111 (51.2)	
Cigarette use before pregnancy				
Yes	110 (37.3)	29 (37.2)	81 (37.3)	0.982
No	185 (62.7)	49 (62.8)	136 (62.7)	
Marijuana use before pregnancy				
Yes	86 (29.2)	26 (33.3)	60 (27.7)	0.343
No	209 (70.8)	52 (66.7)	157 (72.3)	
Family member coresidence				
Partner coresidence				
Yes	145 (49.7)	39 (51.3)	106 (49.1)	0.737
No	147 (50.3)	37 (48.7)	110 (50.9)	
Mother coresidence				
Yes	137 (46.9)	27 (35.5)	110 (50.9)	0.021
No	155 (53.1)	49 (64.5)	106 (49.1)	
Father coresidence				
Yes	39 (13.4)	11 (14.5)	28 (13.0)	0.739
No	253 (86.6)	65 (85.5)	188 (87.0)	
Sibling(s) coresidence				
Yes	100 (34.2)	15 (19.7)	85 (39.3)	0.002
No	192 (65.8)	61 (80.3)	131 (60.7)	
Annual household income				
$0–$4,999	130 (44.4)	39 (50.7)	91 (42.1)	0.082
$5,000–$14,999	68 (23.2)	16 (20.8)	52 (24.1)	
$15,000–$24,999	39 (13.3)	13 (16.9)	26 (12.0)	
$25,000–$34,999	32 (10.9)	8 (10.4)	24 (11.1)	
≥$35,000	24 (8.2)	1 (1.3)	23 (10.7)	
Interpersonal violence				
From partner				
Yes	50 (17.0)	26 (33.3)	24 (11.1)	<0.001
No	45 (83.1)	52 (66.7)	193 (88.9)	
From any family member				
Yes	24 (23.8)	15 (42.9)	9 (13.6)	0.001
No	77 (76.2)	20 (57.1)	57 (86.4)	

**Table 2.** The differences on continuous variables between low and high family functioning groups based on *t* tests (*N* = 295)

Variables	Range	Total mean (±SD)	Low family functioning Mean (±SD)	High family functioning Mean (±SD)	*p*-values
Age (years)	15–21	18.70 (1.63)	18.52 (1.79)	18.76 (1.56)	0.275
Years of education	8–20	11.75 (1.82)	11.61 (2.13)	11.80 (1.69)	0.439
Household size	0–11	3.52 (2.00)	3.74 (2.29)	3.45 (1.89)	0.271
Perceived discrimination	0–5	0.84 (0.81)	1.19 (0.93)	0.82 (0.73)	<0.001
Perceived stress	0–4	1.67 (0.62)	1.94 (0.56)	1.58 (0.62)	<0.001
Perceived social support	0–4	3.13 (0.84)	2.66 (0.11)	3.29 (0.05)	<0.001

SD, standard deviation.

Table 3 presents the unadjusted odds ratios from bivariate logistic regression. Analyses showed that self-identification as Black or Latina (vs. white or other races, OR = 1.79, 95% CI: 0.98–3.28), attending religious services (OR = 1.82, 95% CI: 1.07–3.10), low perceived discrimination (OR = 3.57, 95% CI: 2.84–6.24), low perceived stress (OR = 2.47, 95% CI: 1.45–4.23), and high social support (OR = 3.71, 95% CI: 2.12–6.53) were significantly associated with high family functioning group. Within the family-level factors, annual household income ≥$15,000 (OR = 2.10, 95% CI: 0.98–4.52), mother coresidence (OR = 1.88, 95% CI: 1.10–3.23), and sibling(s) coresidence (OR = 2.63, 95% CI: 1.40–4.94) were associated with high family functioning group; and not experiencing violence from partner (OR = 4.02, 95% CI: 2.13–7.58) or violence from any family member (OR = 4.75, 95% CI: 1.80–12.53) was also associated with high family functioning group.

**Table 3.** Bivariate logistic regression of individual- and family-level factors associated with high family functioning in pregnant young women (*N* = 295)

Variables	Low family functioning *N* (%)	High family functioning *N* (%)	Unadjusted OR (95% CI)	*p*-values
Individual-level factors				
Black or Latina (vs. white and other races)	56 (23.9)	178 (76.1)	1.79 (0.98–3.28)	0.062
Married (vs. single, cohabit, or separated)	9 (22.0)	32 (78.0)	1.32 (0.60–2.92)	0.475
Attending religious services	67 (65.7)	35 (34.3)	1.82 (1.07–3.10)	0.028
Low perceived discrimination	42 (53.9)	36 (46.2)	3.57 (2.84–6.24)	<0.001
Low perceived stress	58 (61.1)	37 (39.0)	2.47 (1.45–4.23)	<0.001
High perceived social support	39 (52.7)	35 (47.3)	3.71 (2.12–6.53)	<0.001
Family-level factors				
Annual household income≥$15,000	169 (71.3)	68 (28.7)	2.10 (0.98–4.52)	0.045
Mother coresidence	27 (19.7)	110 (80.3)	1.88 (1.10–3.23)	0.020
Sibling(s) coresidence	15 (15.0)	85 (85.0)	2.63 (1.40–4.94)	0.001
No violence from partner	26 (52.0)	24 (48.0)	4.02 (2.13–7.58)	<0.001
No violence from any family member	15 (62.5)	9 (37.5)	4.75 (1.80–12.53)	<0.001

Table 4 presents the results of multivariate logistic regression models. With the inclusion of individual-level factors (Model 1), significant associations were observed between attending religious services (adjusted OR = 2.22, 95% CI: 1.20–4.09), low perceived discrimination (OR = 3.04, 95% CI: 1.60–5.75), and high perceived social support (OR = 3.74, 95% CI: 2.01–6.95), and high family functioning. These estimates indicated that young mothers attending religious services, perceiving low discrimination and high social support, were relatively 2.22, 3.04, and 3.74 times, respectively, more likely to have high family functioning.

**Table 4.** Multivariate logistic regression of factors associated with high family functioning in pregnant young women (*N* = 295)

Variables	Adjusted OR	Standard error	95% CI lower	95% CI upper	*p*-values
Model 1 (only individual-level factors)					
Black or Latino	1.13	0.40	0.56	2.27	0.728
Attending religious services	2.22	0.69	1.20	4.09	0.010
Low perceived discrimination	3.04	0.99	1.60	5.75	0.001
Low perceived stress	1.58	0.49	0.85	2.93	0.144
High perceived social support	3.74	1.18	2.01	6.96	<0.001
Model 2 (individual- and family-level factors)					
Black or Latino	0.89	0.57	0.25	3.15	0.855
Attending religious services	2.53	1.51	0.78	8.17	0.121
Low perceived discrimination	2.15	1.28	0.67	6.87	0.196
Low perceived stress	1.99	1.17	0.62	6.32	0.245
High perceived social support	2.53	1.49	0.80	8.02	0.114
Annual household income≥$15,000	9.82	8.87	1.67	56.67	0.011
Mother coresidence	1.37	0.87	0.40	4.78	0.617
Sibling(s) coresidence	2.45	1.72	0.61	9.77	0.206
No violence from partner	2.32	1.64	0.61	9.24	0.213
No violence from any family member	4.94	2.99	1.50	16.21	0.008

OR, odds ratio; CI, confidence interval.

After including both individual- and family-level factors (Model 2), individual-level associations were attenuated and associations between annual household income ≥$15,000 (OR = 9.82, 95% CI: 1.67–57.67, *p* = 0.011) and not experiencing violence from any family member (OR = 4.94, 95% CI: 1.50–16.21, *p* = 0.008) and high family functioning remained significant. These estimates indicated that annual household income ≥$15,000 and not experiencing violence from any family member showed relatively 9.82 and 4.94 times more likely to have high family functioning.

## Discussion

The current study explored individual- and family-level factors associated with family functioning groups among young mothers between 15 and 21 years of age. As an important aspect of family resilience, high family functioning can give these families the ability to make positive adaptation when facing the stress of pregnancy. Families with high family functioning are equipped with good communication, positive affect, and problem-solving processes, and perceiving their families as an important source of support. High family functioning can be significant for young mothers' psychological wellbeing, with studies showing that women living in families with high family functioning are less likely to have emotional symptoms and are more likely to have higher levels of self-efficacy in maternal roles.^37–39^ Based on this information, it might be beneficial that young mothers count on significant family members to gain positive experiences during pregnancy.

Regarding individual-level factors, consistent with prior research conducted with adult mothers,^13^ our results found a significant association between higher social support and higher family functioning. This finding highlights the significance of social support in cultivating family functioning among young pregnant women. Having a support network to share resources is one of the most critical factors in determining an individual's and family's ability to acquire coping resources and skills in the face of stress.^40^ Social support can increase the sense of competence or control against challenging circumstances.^40^ Childbearing families with young mothers may face multiple challenging situations and thus may need additional support and resources for personal transformation.

Similarly, attending religious services (e.g., church, temple) was found to be associated with higher family functioning. Research suggests that religiosity gives families an optimistic attitude toward adversity, a shared value to seeking purpose in faith, and the ability to understand and overcome stressful situations.^26^ Additionally, membership in religious services is often an avenue to gain extended supportive networks for family members.^12,26,41^ Thus, it is possible that attending religious services may provide young mothers with feelings of belonging and closeness in the communities and help them to develop a sense of belonging in their families.

Moreover, lower discrimination was associated with higher family functioning. Thus, when individual family members experience less external stressors like discrimination, they may be more likely to have more positive family interactions and more satisfying relationships with their family members.^42,43^ It is possible that external stress (e.g., structural discrimination) has great power on changing the individual development and family functioning. Therefore, strategies to mitigate the negative impact of discrimination (from race, income, physical appearance, and pregnancy at young maternal age) might be helpful to young mothers' family lives. The larger social contexts (e.g., schools and communities) might pay attention to different sources of discrimination that pregnant young women experience. For example, schools could provide resources for their students to learn about the stress and significance of pregnancy.

While most of our results on significant individual-level factors are consistent with prior research,^13^ there have been some interesting findings in which some factors (e.g., lower stress/anxiety) were not associated with higher family functioning among young mothers. First, our findings showed no significant association between general stress and family functioning. For pregnant young women, different types of stress can be complicated, such as experiences of discrimination, interpersonal pressure at school or work, anxiety about becoming a new mother, and limited financial resources. Rather than focusing on the general stress level, it is possible that the specific type of stress (e.g., high discrimination) exposes the risk for family dysfunction.

Regarding family-level factors, consistent with the conceptual review of family resilient factors,^26^ our results showed that how a family is structured, indicated by annual household income and violence in the family system, contributes to the family's capacity to be high functioning. Specifically, annual household income≥$15,000 was associated with higher family functioning. Providing for the needs of its members is the primary task of a family unit, leading to more positive interactions between family members.^41^ Adequate household income allows families to provide sufficient economic support to pregnant young women, which is needed to meet the increasing demands of women during pregnancy (e.g., nutrition care) and following birth (e.g., feeding the new baby). In addition, access to adequate resources may provide a sense of security for families. As such, young mothers need to know that their families will have the financial strength to protect them against an unexpected crisis.

Lastly, less interpersonal violence was associated with higher family functioning. Interpersonal violence in a family unit indicates a strong source of stress and an imbalance of power between family members, which exposes risk to the psychological functioning of individuals and the shared decision-making process in families.^44^ Pregnancy is a unique opportunity for health care providers to identify and screen for any interpersonal violence. Building the relationship with the client is a longitudinal process involving repeated contact during visits and offering a great opportunity to develop trust between the woman and members of the health care team.^45^ Besides obvious warning signs, providers should be tuned to other more discrete clues (e.g., medical history, the patient's and the partner's behavior, physical examination) that may indicate violence.

## Research and Clinical Implications

The current study has one important implication for future studies. We included individual- and family-level factors affecting family functioning based on prior review; however, there may be novel factors that apply to pregnant young women population as they are facing many developmental challenges such as the desire for self-identification and the transition to parenthood earlier. We suggest future studies need to consider the individual psychosocial factors that are unique to pregnant young women when exploring the concept of family functioning during the perinatal period, such as peer pressure, body anxiety, and parenthood expectation.

Our study has implications for clinical maternity care. While the clinical practice tends to focus on the mother herself and her health, the fact that pregnant young women perceived the positive effect of their families could be significant. The model of care should be structured to support the continuity of care, in which health care providers have the opportunity to develop ongoing relationships with pregnant women and their families.^13,46^ Through assessment of and conversations around family functioning, health care providers could discover the specific strengths of each family and engage significant family members with whom the young woman has positive interactions and relationships. Families with lower family functioning should be offered the opportunity (e.g., providing resources for family activities and referring to family therapies) to develop coping mechanisms and strengthen bonds within the family unit.^13^

Moreover, given that families are within the larger social contexts, the bonds with the external environment (e.g., the community and school) can be a good way to intervene. Schools and community agencies should provide resources, support, and outreach for childbearing families with young mothers. If the family unit shares religious beliefs, expanding the support network through religious services could also be a good choice.

### Limitations

Several study limitations should be noted. The current study was a secondary data analysis aiming to explore factors associated with family resilient outcome among young mothers. Therefore, our exploratory factors were limited to variables collected in the original dataset. For example, other aspects of family resilience, such as shared family belief systems, should be considered to picture family resilience in the stress of pregnancy comprehensively.

Additionally, most young mothers in our sample identified as Black or Latina and reported low household income, which limits the generalization of our study results to other women. Another concern about the generalizability of the current result may come from the fact that this sample recruited pregnant young women being in a romantic relationship at baseline. This makes the sample different from many young women who are not in romantic relationship with the biological fathers of the focal baby. While the concept of family functioning applies to these young women as well, it can be interesting to examine the difference of family functioning between young women who have a romantic relationship during pregnancy and who do not have.

Lastly, the measure of family functioning (Family Functioning Scale) should be explored to generate a standardized cutoff point for low or high family functioning groups, for example, comparing FFS with other family functioning scales and applying FFS across diverse samples.

## Conclusion

The current study found that young women who ever attended religious services, perceived low discrimination and high social support, were more likely to live in families with higher family functioning. At the family level, adequate household income and never experienced violence in the family system were also associated with higher family functioning. During antenatal visits, health care providers are well positioned to discover and utilize each family's strengths to provide the optimal caring experience for young mothers and their families as a unit.

## Data Availability Statement

Data from the PARTNRS project could be attained through an application submitted to Dr. Kershaw, Yale University.

## Ethics Approval Statement

This secondary data analysis did not involve any follow-up data collection with participants.

## Abbreviations Used

CI: 95% confidence interval
FFS: Family Functioning Scale
OR: odds ratio
